# Proton Magnetic Resonance Spectroscopy (^1^H-MRS) Reveals Geniculocalcarine and Striate Area Degeneration in Primary Glaucoma

**DOI:** 10.1371/journal.pone.0073197

**Published:** 2013-08-29

**Authors:** Yan Zhang, Xiuyu Chen, Ge Wen, Guijun Wu, Xuelin Zhang

**Affiliations:** 1 Zhongshan Ophthalmic Center, State Key Laboratory of Ophthalmology, Sun Yat-sen University, Guangzhou, GuangDong, China; 2 Department of Radiology, Nan Fang Hospital, Southern Medical University, Guangzhou, GuangDong, China; 3 Department of Ophthalmology, Nan Fang Hospital, Southern Medical University, Guangzhou, GuangDong, China; Universidade Federal do Rio de Janeiro, Brazil

## Abstract

**Background:**

Glaucoma is a collection of neurodegenerative diseases that affect both the retina and the central visual pathway. We investigated whether metabolites' concentrations changed in the geniculocalcarine (GCT) and the striate area of occipital lobe by proton magnetic resonance spectroscopy (^1^H-MRS), suggesting neurodegeneration of the central visual pathway in primary glaucoma.

**Methodology/Principal Findings:**

20 patients with glaucoma in both eyes were paired with 20 healthy volunteers in same gender and an age difference less than 3 years. All the participants were examined by MR imaging including T_1_ Flair, T_2_ FSE and ^1^H-MRS. The T_1_ intensity and T_2_ intensity of their GCTs and striate areas were measured. The ratio of N-acetylaspartate (NAA)/Creatine (Cr), Choline (Cho)/Cr, glutamine and glutamate (Glx)/Cr were derived by multi-voxels ^1^H-MRS in the GCT and the striate area of each brain hemisphere. The T_1_ intensity and T_2_ intensity had no difference between the groups. Significant decreases in NAA/Cr and Cho/Cr but no difference in Glx/Cr was found between the groups in both the GCT and the striate area.

**Conclusions/Significance:**

Primary glaucoma affects metabolites' concentrations in the GCT and the striate area suggesting there is ongoing neurodegenerative process.

## Introduction

Glaucoma is the second common cause of irreversible blindness worldwide [1]. It is essentially a collection of neurodegenerative diseases that affect both the retina and the central visual pathway, leading to degenerative changes in the lateral geniculate nucleus, geniculocalcarine (GCT)and visual cortex [2]–[13]. Glaucoma results in retinal ganglion cell (RGC) axon degeneration and death. Patient with glaucoma has a typical excavation at the optic nerve head, neuroretinal rim thinning, sectoral retinal nerve fiber layer defects with progressive retinal visual field defect. A number of mechanisms have been invoked to explain the mechanism of glaucoma, including reactive oxygen species, excitotoxicity, defective axon transport, trophic factor withdrawal and loss of electrical activity [12]. These pathophysiologic actions lead to a serious of biochemical compounds changes in brain tissue.

Proton magnetic resonance spectroscopy (^1^H-MRS) is a non-invasive technique to detect and quantificate certain biochemical compounds, like N-acetylaspartate (NAA), Creatine (Cr), Choline (Cho), glutamine and glutamate (Glx) in brain tissue. NAA is a major marker for neuronal integrity. It is particularly localized within neurons and related to neuronal processes. Its decrease is routinely an indicator of neuronal loss or dysfunction. Cr, which plays an important role in energy metabolism, has been reported to be constant throughout the brain and resistant to change in several degenerative brain diseases [14]. Cho is the total amount of cytosolic choline. Its decrease suggests the reduction of cell density, the delay of cell renewing and the damage of signal transport system in neuron [15]. Trans-synaptic damage caused by excitotoxicity of Glx is an important mechanism of glaucomatous central visual pathway injury.

However, only a few researches concerned to investigate glaucoma with the ^1^H-MRS [14]–[16]. Boucard et al. measured the absolute concentrations of NAA, Cr, and Cho in the striate area using single-voxel^1^H-MRS in patients with progressive visual field defects (seven patients of age-related macular degeneration (AMD) and seven patients of primary open-angel glaucoma (POAG)) and a control group. The results indicated no significant differences between the patients and the control group.

In this paper, we studied if the concentrations of the metabolites NAA, Cr, Cho and Glx in the GCT and the striate area were affected by primary glaucoma using multiple-voxels^1^H-MRS. We detected significant decreases in NAA/Cr and Cho/Cr but no difference in Glx/Cr in the GCT and the striate area among the glaucoma and the control groups. This result suggests the ongoing neurodegenerative process in the central visual pathway of primary glaucoma.

## Materials and Methods

### Ethics Statement

The research protocol was approved by the ethics committees for clinical research at Southern Medical University and Sun Yat-sen University. All of the procedures involving the participants were conducted following the Declaration of Helsinki and institutional guidelines in compliance with the stated regulations. Written informed consent was obtained from all of the participants.

### Subjects

Subjects with glaucoma were recruited from the Department of Ophthalmology, NanFang Hospital, Southern Medical University (Guangzhou, China) and ZhongShan Ophthalmic center, Sun Yat-sen University (Guangzhou, China).The group consisted of 20 patients suffering from primary glaucoma in both eyes (eight female and twelve males; mean age 45 years, range 19–76). All were diagnosed according to the “Clinical practice guidelines of glaucoma in China” [17]–[18]. These patients varied from the early stage to advanced glaucoma. There were 8 primary open-angle glaucoma (POAG) and 12 primary angle-closure glaucoma (PCAG). Patients with any other disease that might affect intraocular pressure and visual field were excluded. The excluding criteria includes iridocyclitis, ocular trauma, disorders of optic nerve (affection, inflammation, ischemia and pressure-induced optic atrophy), AMD, diabetic retinopathy, pituitary tumor, other general health problem and drug and/or alcohol abuse.

For the control group, 20 healthy subjects (eight female and twelve males; mean age 45 years, range 20–78) were recruited from the examinees in the same apartments above. The examinees were paired with the patients with the same gender and an age difference less than 3 years. Control subjects were required to have the best corrected visual acuity>0.8, without visual field defect and any other ophthalmic and neurologic disease. No history of drug and/or alcohol abuse.

### Materials and data acquisition

**Figure 1:**
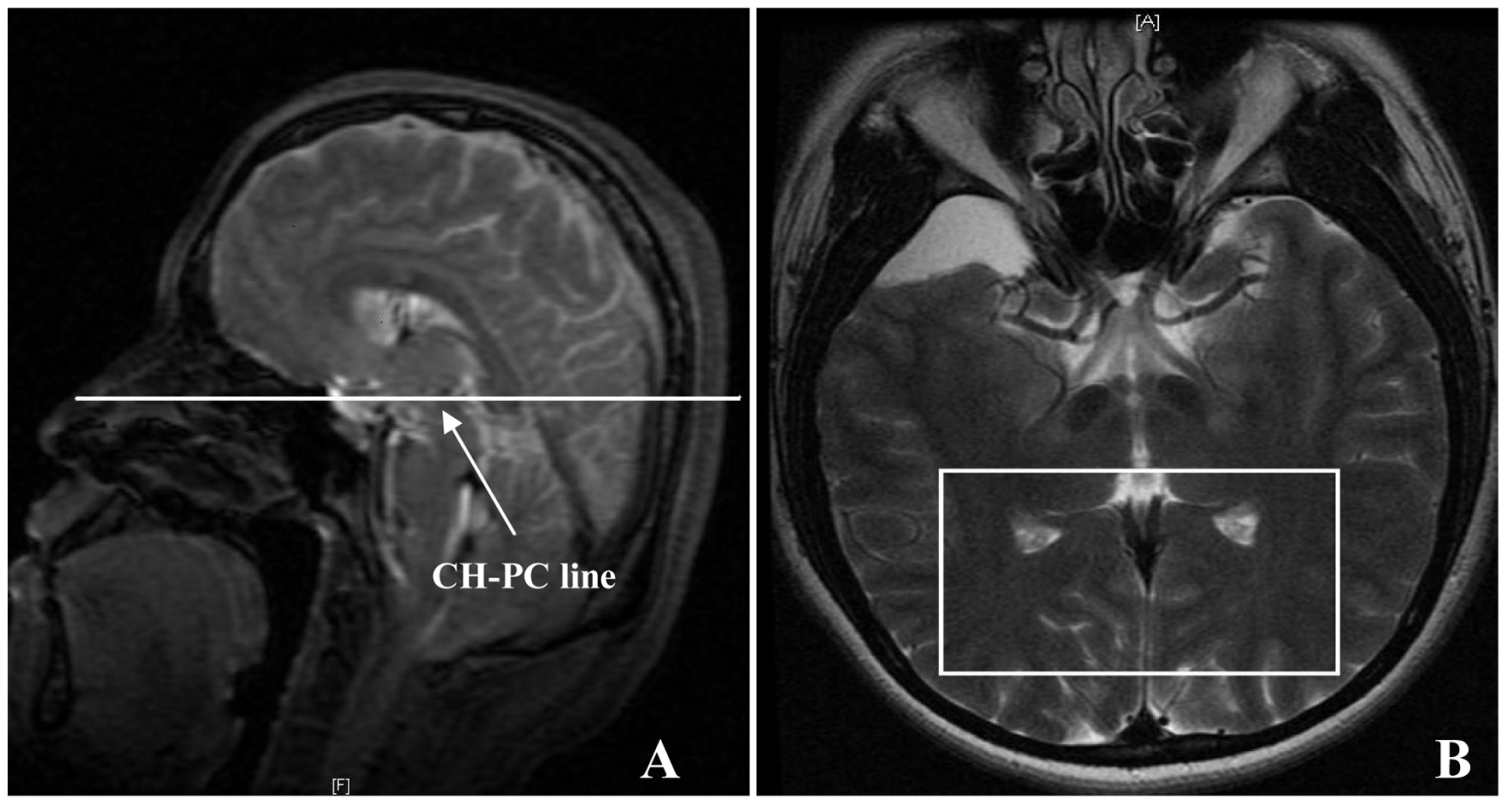
The volume of interest in multi-voxels 2D-^1^H-MRS. Figure 1A was the T_2_WI image of the median sagittal plane. The subject kept the lower jaw upwards to make the CH-PC line horizontal. Figure 1B The VOI (white box), including bilateral GCTs and the striate area, located in the axial T_2_WI image of the median sagittal plane.

MRIs were performed using a whole body 3.0 Tesla scanner (SIGNAL EXCITE, GE, USA) with an 8-channel head-phased array coil. The subject was in supine position with head inside the array coil. Head movement was limited by plastic sponge fixation cushions. The lower jaw of the subject kept upwards to let the chiasmatic-posterior commissural (CH-PC) line stay horizontal. (Figure 1A) The surface mark was the ligature from one ala nasi to the ipsilateral external auditory canal. The position line was the ligature of both external canthus.

All subjects underwent routine brain MRI, which included T_1_WI Flair, T_2_WI FSE, T_2_ WI Flair and DWI sequences. Consecutive slices in identical locations for all sequences were acquired with a slice thickness of 5 mm. The parameters of T_1_WI-FLAIR: TR = 2580 ms, TE = 24 ms,slice thickness of 10 mm, inter-slice separation 0 mm,matrix size320×256,FOV 240×180 mm, NEX = 2.The parameters of T_2_WI: TR = 5100 ms, TE = 138 ms, slice thickness of 10 mm, inter-slice separation 0 mm, matrix size 288×224, FOV 240×240 mm, NEX = 1.

The parameters of DWI: TR = 6200 ms, TE = 87.1 ms, b = 1000 s/mm^2^, slice thickness of 10 mm, inter-slice separation 0 mm, matrix size192×192, FOV 240×240 mm, NEX = 2. Cross section scanning is the routine, some with coronal section and sagittal section added.

Multi-voxels ^1^H-MRS was performed by the point resolved spectroscopy (PRESS) using the single –channel quadrature headcoil. The parameters of ^1^H-MRS: TR = 2000 ms,TE = 35 ms, voxel thickness of 10 mm, spacing 10 mm, FOV 18×18 cm, NEX = 1. The volume of interest (VOI) was located in the layer of the CH-PC line or the layer above. The location of 1H-MRS was achieved in axial T_2_WI images with bilateral GCTs and the striate area. The VOI was mobilized to include bilateral GCTs and the striate area as far as possible while avoiding the inclusion of skull, fat and air sinus. (Figure 1B) Six VSS were placed around the VOI to reduce the partial volume effect of surrounding tissues. ^1^H-MRS scanning started when the FWHM ≤10, the water suppression (WS) ≥98%. The total scan time was 8 min 6 s.

### Data Analysis

**Figure 2:**
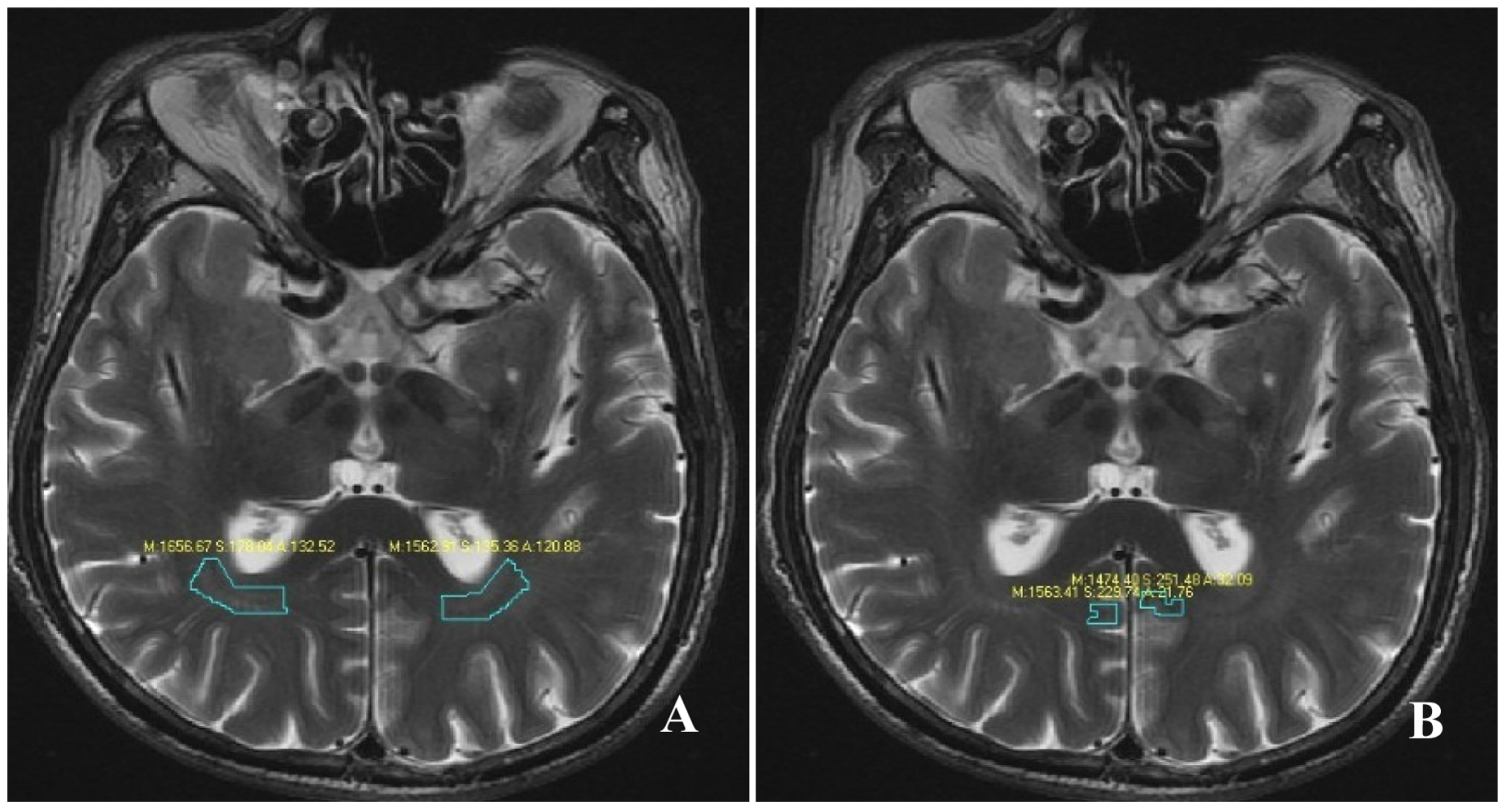
Regions of interest in the measurement of T2 intensity. Figure 2A displayed the ROIs of the GCTs, while Figure 2B displayed the ROIs of the striate area.

Measure the T_1_ intensity and T_2_ intensity of the GCTs regions of interest (ROIs) and the striate area ROIs in T_1_ Flair and T_2_WI FSE images. Each location was measured for three times to get the mean value. (Figure 2).

**Figure 3:**
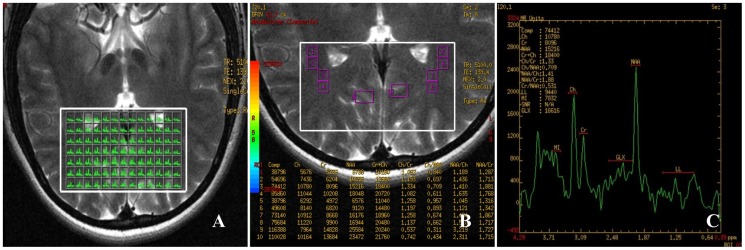
Example of region of interest and spectra. Figure 3A displayed all the fixed-voxels in the VOI (white box). The size of each voxel was 31.6 mm^2^, as recommended by the software. Figure 3B was the amplification of Figure 3A. Select several individual voxels as the ROIs in bilateral GCTs and the striate area. ROI 1 to ROI 8, all of which were individual voxels, distributed symmetrically in bilateral GCTs. ROI 9 and ROI 10 located in the striate area, each contained six voxels. Figure 3C was the spectra of ROI 2 in the right GCT, with NAA and Cho peaks.

The multi-voxel ^1^H-MRS data and T_2_WI images simultaneously were post processed using the scanner software “FunctTool”. Four types of pictures: chemical shift image (CSI), spectral image (SI), metabolic image (MI) and metabolic-anatomical diagram integration image (AI+MI) were obtained. Choose four fixed-voxels with high signal noise ratios (SNR) as ROIs on each GCT in the AI+MI. The spectroscopy curves of these voxels had straight baseline and significant narrow peaks. Take the averaged value of the voxels as the result. The ROI of unilateral striate area consisted of 6 voxels and took the averaged value of the voxels. (FIigure 3A, B) The concentrations of metabolites including NAA, Cho, Cr and Glx were measured. The results were recorded as NAA/Cr, Cho/Cr and Glx/Cr. (Figure 3C).

### Statistics

Independent-Samples T Test was used to evaluate the differences in T_1_ and T_2_ intensities and concentrations of metabolites between the glaucoma group and the control group. p<0.05 was used to determine statistical significance. All analyses were performed using SPSS 13.0 software (IBM SPSS, USA).

## Results

There was no abnormal finding in routine MR imaging in either group. The T_1_ intensities of the GCTs were 1788±213 and 1785±213 while the T_2_ intensities were 1232±126 and 1224±126 respectively for the glaucoma group and the controls. No difference existed between the groups (t = 0.051, P = 0.959;t = 0.190, P = 0.850). The T_1_ intensities of the striate areas were 1479±198 and 1469±184 while the T_2_ intensities were1447±158 and 1433±161 respectively for the glaucoma group and the controls. There was no difference between the groups (t = 0.164, P = 0.870; t = 0.277, P = 0.783).

**Figure 4:**
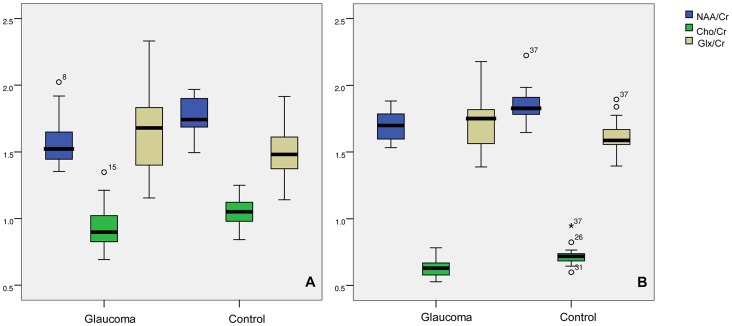
Metabolite concentrations. Figure 4A displayed the averaged concentrations ratios of NAA, Cho and Glx to Cr for the glaucoma group and the controls in the GCTs. Figure 4B displayed the averaged concentrations ratios in the striate area. The Independent-Samples T Test between the two groups showed significant differences for the NAA and Cho concentrations but no difference for the Glx concentration.

Boxplots in figure 4A depicted the averaged concentrations of NAA, Cho and Glx in the GCTs. In the GCTs, independent-samples t test between the glaucoma group and the controls showed significant decreases in the NAA and Cho concentrations. For the NAA/Cr: t = 4.120, P = 0.000; the Cho/Cr: t = 2.267, P = 0.029. But there was no significant difference in the Glx concentertion, for the Glx/Cr: t = 1.926, P = 0.064.

Boxplots in figure 4B depicted the averaged concentrations of each metabolite in the striate areas. The independent-samples t test between the glaucoma group and the controls showed significant decreases in the NAA and Cho concentrations. For the NAA/Cr: t = 3.047, P = 0.004; for Cho/Cr: t = 4.858, P = 0.000. But there was no significant difference in the Glx concentertion, for the Glx/Cr: t = 1.799, P = 0.081.

## Discussion

There is no difference between the glaucoma group and the control group in T_1_ intensity and T_2_ intensity of the GCTs and the striate areas. However, in the glaucoma group, both the NAA/Cr and Cho/Cr were reduced in the GCTs and the striate areas comparing with the control group. But the Glx/Cr was stable. Cr is mainly composed of creatine and creatine phosphate. Its concentration is relatively stable either in normal or pathological brain tissues according to previous researches. Hence Cr is often taken as the reference for elevating the changes of other metabolites. Our results indicate that glaucoma does induce a measureable decrease in NAA and Cho metabolic concentration in the GCT and the striate area while the Glx concentration remains unchanged.

The NAA concentrations decreased significantly in the GCT and the striate areas in the glaucoma group. The NAA represents the survival of neurons and axons. Functional abnormity or structural damage of neurons and axons will cause its reduction. The reduction of NAA concentration reflects the neuronal damage and axonal lost accurately and can be found in many kinds of central neurodegenerative diseases [3]–[5]. In glaucoma, initiating factors like elevated IOP leads to a blockade of the normal shuttling of cytoplasmic cargoes up and down the axon, which lead to the death of retinal ganglion cells. Neurotrophic survival and growth signals from the retina, the optic nerve and their targets in the brain also determinate the survival of retinal ganglion cells. Retinal ganglion cells may also become less responsive to trophic factors after injury, possibly as a result of decreased electrical activity after optic nerve injury [12].

The death of the ganglion cells may cause a serious of negative feedback from the optic nerve to the central visual pathway, which finally cause the neuronal damage in the central visual pathway. Reduced NAA concentrations only can be measured when degenerative processes are currently taking place [12]. All the patients were in the attack stage in our research. We could conclude that in the attack stage, there were neuronal damage and axonal lost in the GCTs and the striate areas.

Cho indicates the total amount of choline in brain tissue, 98% of which presents as phosphorycholine (PCho), glycerophosphorycholine (GPC) and phosphatidylcholine. Cho participates in the synthesis and degradation of cell membrane. It marks the disintegration of myelin sheath. Decreases in Cho levels are related to apoptosis, the damage of signal transduction system in neurons and the delay of cellular metabolism [14].

The decrease in Cho reveals that neuron apoptosis in the GCTs and the striate areas in the glaucoma group. Even without knowing the underlying molecular or cellular mechanism by which RGCs are initially damaged in glaucoma, considerable progress has been made in understanding the downstream pathways that lead to RGC dysfunction and death. Ultimately, RGCs primarily die in glaucoma by apoptosis. Apoptosis is the process of programmed cell death resulting in sequential degradation of intracellular organelles [12]. Apoptosis can be militated by “intrinsic pathways” that are activated after the loss of pro-survival signals from neighboring cells in the brain [12]. Apoptosis of a great number of neurons have already been found out in many neurodegenerative diseases, such as the Alzheimer disease and the Parkinson disease. Cho is one of the important substances to regulate the apoptosis of cells. The deficiency of Cho is a latency to induce the apoptosis of neurons. DNA fragmentation is the typical presentation of apoptotic cells. The lack of Cho makes DNA fragmentation appear in early stage. Therefore, the reduction of Cho in the GCTs and the striate areas of glaucoma patients suggest the apoptosis of neurons in these regions.

Cho is the basic material to synthesis the acetylcholine (Ach). *A*ch is an important exciting neurotransmitter in the synaptic transmission. It is also an indispensable transmitter in the transduction of visual signals. The release of Ach is the symbol of visual center activating after the visual stimulation [19].

In addition, the Ach receptors are stimulated by the afferent visual signals and the visual center together [20]. Therefore both the visual field defect and the damage of visual pathway can induce the down-regulation of the Ach receptors and the reduction of synthesis and release of Ach [21].

The damage of GCT and their axons caused by intraocular pressure elevation and abnormal blood perfusion may reduce the release of Ach in the central visual pathway of glaucoma. As it dose, the trans-synaptic damage occurs. The afferent visual signal is reduced, followed by the decrease of synthesis and release of Ach. The demand for Cho decreases as well.

Choline acetyl transferase (ChAT) is the key enzyme in the ACh synthesis. ChAh mainly delivers by means of anterograde axonal transport. The blockage of anterograde axonal transport behind optic papilla has been certified in glaucoma patients [22]. The lack of ChAT will cause synthesis disorder of ACh and down-regulate the storage of Cho in visual center. For instance, the release of Ach decreases in contralateral visual center six weeks after the induction of monocular primary open angle glaucoma in the rat model [14].

Cho is the basic material to synthesis the phosphatidylcholine (PtdCho) also.

PtdCho is the important composition to construct the lipid bilayer of cholinergic neuron. The reasons why Cho deficiency will induce neuron apoptosis have two aspects. On one hand, the decrease of PtdCho density will damage the cellular membrane, mitochondrial membrane and lysosome membrane. The damage of these membranes will cause the dysfunction and metabolic disorder of cell and trigger the prestored apoptosis process [23]. On the other hand, the damage of axons will cause the blockage of anterograde axonal transport and retrograde axonal transport. The secondary deficiency of trophic factors, growth factors, ischemia and anoxia will slow down or stop the metabolism of cell membrane and cause the decrease of Cho. Glaucomatous blockage stops retrograde transport of neurotrophic factors, such as brain derived neurotrophic factor (BDNI) and other neurotrophic factors. Some researches in recent years found out that exogenous citicoline can enhance the reactivity of visual center in glaucoma patients [24]. The mechanism of this phenomenon is not completely clear. One hypothesis is that the citicoline translates to Cho and cytidine rapidly and enters into the central nervous system from the blood brain barrier. Babb et al. found out that take citicoline orally or intake Cho directly will increase the concentration of Cho in brain tissues by ^1^H-MRS [25]–[26]. The neuroprotective effect of exogenous citicoline may relate to the supplement of intracephalic Cho deficiency. It may also increase the synthesis of Ach, dopamine and noradrenaline.

Glx is one of the most important neurotransmitters in the brain tissue of mammals. Glx has the highest content in cortex, sea horse and cerebral. Glx participates in the metabolism of intracephalic ammonia. It is the precursor of inhibitive neurotransmitter–aminobutyric acid. The concentration of glx increases in the ischemia, anoxia and hepatic encephalopathy [27]. Trans-synaptic damage caused by oxidative stress reaction and glutamate excitotoxicity is the important mechanism of central visual pathway damage in glaucoma. Intraocular pressure elevation and hypoperfusion cause the blockage of anterograde axonal transport and retrograde axonal transport in optic nerve fibers. These actions are followed by the suspending of target-derived neurotrophic factors along with massive excitotoxins [28]. Increased reactive oxygen species and decreased concentrations of antioxidants have been found in the glaucomatous vitreous, as have oxidative DNA damage and oxidative alterations of the trabecular meshwork [12].

Excitotoxicity is thought to occur when dying cells release excessive amount of neurotransmitters such as glutamate. In glaucoma, the uptake of Glux is decreased in neurons with the weakening of visual stimulation. The concentration of Glx in extracellular matrix increases. The high concentration of Glx induces the hyperactivation of N-methyl-D-aspartate(NMDA) sensitive glutamate channels in neurons. Hyperactivation of NMDA sensitive glutamate channels in neurons may lead to a deleterious increase in intracellular calcium, activation of nitric oxide synthase resulting in nitric oxide production, and other metabolic dysregulation, injuring these adjacent neurons in a secondary or bystander death. Activation of intracellular calcium-activated proteins (e.g., calcineurin) and calpain and proteases increased expression of pro-apototic genes, and down regulation of anti-apoptotic genes lead to programmed cell death. Whether glutamate is directly or indirectly toxic to neurons, blocking excessive glutamate activation remains an area for investigation [29]–[30].

In Boucard et al. 's research, ^1^H-MRS revealed normal metabolite concentrations in glaucoma, suggesting that there is no ongoing occipital degeneration. They discuss the possibility that the absence of a reduction in NAA concentrations compared to controls most likely indicates that no degeneration is currently occurring in the occipital region of glaucoma patients. Glaucoma progresses at a very slow rate, which may prevent detectable NAA changes [31]. On the other hand, ROI defined by single-voxel may not completely cover a degenerated region. Chan et al. revealed choline reduction in the visual cortex by ^1^H-MRS in an experimental rat model of chronic glaucoma in 2009 [14]. But the NAA/Cr was not reduced, which they attribute to the slow progresses of glaucoma also.

In conclusion, reduced NAA and Cho absolute concentrations but no significant difference in Glx concentration are found in the GCTs and the striate area of patients suffering from primary glaucoma, suggesting there is ongoing neurodegenerative process. The application of ^1^H-MRS for the metabolic evaluations in vivo is an important progress in exploring the mechanisms of glaucoma. Even without knowing the underlying molecular or cellular mechanism by which RGCs and neurons in the central visual pathway are initially damaged in glaucoma, the downstream pathways of their dysfunction and death are gradually clear. Ultimately, the RGCs and neurons primarily die by apoptosis in glaucoma, which certified that primary glaucoma is a degenerative disease from the retina to the central visual pathway.
